# Sensing Pressure Distribution on a Lower-Limb Exoskeleton Physical Human-Machine Interface

**DOI:** 10.3390/s110100207

**Published:** 2010-12-28

**Authors:** Stefano Marco Maria De Rossi, Nicola Vitiello, Tommaso Lenzi, Renaud Ronsse, Bram Koopman, Alessandro Persichetti, Fabrizio Vecchi, Auke Jan Ijspeert, Herman van der Kooij, Maria Chiara Carrozza

**Affiliations:** 1 ARTS Lab, Scuola Superiore Sant’Anna, viale Rinaldo Piaggio 34, 56025, Pontedera (Pi), Italy; E-Mails: n.vitiello@sssup.it (N.V.); t.lenzi@sssup.it (T.L.); a.persichetti@arts.sssup.it (A.P.); f.vecchi@sssup.it (F.V.); carrozza@sssup.it (M.C.C.); 2 Biorobotics Laboratory, Institute of Bioengineering, Ecole Polytechnique Fédérale de Lausanne (EPFL), CH-1015, Lausanne, Switzerland; E-Mails: renaud.ronsse@epfl.ch (R.R); auke.ijspeert@epfl.ch (A.J.I.); 3 Biomechanical Engineering Laboratory, Institute for Biomedical Technology and Technical Medicine (MIRA), University of Twente, 7500 EA Enschede, The Netherlands; E-Mails: B.Koopman@ctw.utwente.nl (B.K.); h.vanderkooij@utwente.nl (H.K.)

**Keywords:** human-robot interaction, physical human-machine interface, distributed force sensor, lower-limb exoskeleton

## Abstract

A sensory apparatus to monitor pressure distribution on the physical human-robot interface of lower-limb exoskeletons is presented. We propose a distributed measure of the interaction pressure over the whole contact area between the user and the machine as an alternative measurement method of human-robot interaction. To obtain this measure, an array of newly-developed soft silicone pressure sensors is inserted between the limb and the mechanical interface that connects the robot to the user, in direct contact with the wearer’s skin. Compared to state-of-the-art measures, the advantage of this approach is that it allows for a distributed measure of the interaction pressure, which could be useful for the assessment of safety and comfort of human-robot interaction. This paper presents the new sensor and its characterization, and the development of an interaction measurement apparatus, which is applied to a lower-limb rehabilitation robot. The system is calibrated, and an example its use during a prototypical gait training task is presented.

## 1. Introduction

Exoskeleton and wearable robots have seen a huge expansion in their application field in the last decade [1], even though research in this field started in the sixties [2]. They are now applied to several fields, including power augmentation for the military [3] or medical assistance [4], rehabilitation [5] and in haptic interfaces [6]. A distinctive characteristic of exoskeletons compared to other robotic interfaces with haptic feedback is their close physical and cognitive coupling between the robot and the user [7]. The components—physical and control—that allow this physical and cognitive cooperation constitute the human-robot interface. In this work, we are interested to physical human-robot interfaces, *i.e.*, the mechanical and sensory components that mediate the transfer of physical interaction between the user and the exoskeleton [8].

There are two widespread ways to interface wearable robots with the user: connection cuffs and orthoses. Connection cuffs are soft belts of adjustable size that are fastened to the user’s limbs: one cuff is used for each connection point. An example of this solution is adopted in the Lokomat® exoskeleton [5], as well as in the LOPES lower-limb exoskeleton [9]. Similar solutions are adopted in other upper-limb exoskeletons, such as the ESA Human Arm Exoskeleton [10], the Dampace [11]-or the Armin II [12], and lower-limb exoskeletons like Alex [13] and HAL [4]. Orthoses, on the other hand, are shells made of plastic or other orthopedic materials which can be worn on the part of the limb onto which the rehabilitation robots apply forces. They have been used in ankle [14], knee and lower limb [15,16], and upper limb [12,17] exoskeletal robots. Both solutions increase the human-robot interaction area and, therefore, improve comfort, ergonomy and safety of the robot. This is of particular interest in rehabilitation robots, where lower pressure values increase the overall acceptance and usability of the robot-mediated therapy [18]. The robotic device transfers loads to the user’s limbs by providing joint torques, and transferring them to interaction forces at the attachment points with the user. This contact force load is then distributed on the physical interface and finally results in a pressure distribution on the user’s skin. State of the art of exoskeleton robots shows two different ways to quantify physical human-robot interaction: by directly measuring interaction force, or through and estimation of interaction torque.

The estimation of interaction torque transferred from the robot to the user can be made by measuring the torque exerted by the robot joint, and by removing the inertial, Coriolis, friction and gravity torque components needed to move the robot. The remaining torque is that transferred to the user through the physical interface. The robot torque can be measured through a torque sensor, or, when using series elastic or other compliant actuators, by an equivalent measure of the deformation of the linear elastic element, as in the LOPES [9]. The accuracy of the dynamic and friction model of the robot is critical to get reliable interaction measurements, and is notably difficult to obtain. Another criticality relies in the presence of interaction dynamics, e.g., due to the presence of soft tissues and compliant physical interfaces (such as belts).

Estimation of interactive torque can be used to compute how loads are transferred to the physical interface with the user. To do that, one needs a model of the connection and interaction between the robot and the user, to correlate the torques measured at the robot joint with interaction forces at the attachment points. This model may be difficult or even unfeasible to obtain, especially when multiple attachments are used for each link, or when orthotic interfaces are applied.

An alternative approach is that of directly measuring the interaction force at the attachment points. This can be retrieved through a load cell, placed at the connection between the cuff/orthosis and the exoskeleton link, such as in the ESA Human Arm Exoskeleton [10] or in the Alex [13], or by evaluating the deformation of an elastic transmission element, as in the MIT leg exoskeleton [19]. An equivalent method is that of measuring the deformation of the structure of the robot links, as in the HAL suite [4].

Force measurements, however, have some drawbacks to be considered. First of all, they hide the information related to the distribution of pressures at the cuff/orthosis. This information can be extremely useful, being directly related with the safety and comfort felt by the user during the robot operation: high (peak) pressures might be uncomfortable or even painful to the user [20,21], and may impact the safety and effectiveness of the rehabilitation therapy [18]. Moreover, when belts are used to strap the user to the device, such as in [5], the forces distributed on the belt may compensate each other and, therefore, not result in a measurable force at the connection point, while effectively loading the user’s skin. This is the case, for example, when the belt is fastened, and consequently applies a “preloading” pressure to the limb. Finally, load cells cannot be used when the interaction between the user’s limb and the robot link is not mediated by a finite number of attachments, but by a distributed area, as in the case of powered orthoses like [17].

For these reasons, it seems natural to measure a distributed interaction force using a distributed measurement system in contact with the wearer’s limb, where the interaction actually takes place. A solution of this kind could involve the use of a thin, distributed pressure sensor, to be inserted between the user and the cuff/orthosis interface, covering the whole interaction area. Ideally, applying such a sensory system should not require design changes in the device, to make it applicable to any kind of robot. Furthermore, a local sensorization placed in contact with the limb would measure exactly what the user is feeling on his limb, and would allow for a real assessment of the comfort of the interaction.

In this work we propose a novel application of distributed force sensing to monitor human robot interaction in exoskeletons. To obtain this measure, we developed a new force sensor, based on an opto-electronic transduction principle, specifically adapted to the requirements arising from the human-robot interaction application (*i.e.*, soft material, force range, size, number of sensors). This sensor is loosely based on an existing tactile sensing technology developed in our laboratory, the Skilsens technology, which we adapted for this purpose. A prototype for a new sensory system was developed, and tested on the attachment points of a lower-limb exoskeleton, the LOPES gait rehabilitation robot. This sensory system represents a first step towards the development of a general-purpose, flexible and adaptable distributed interaction measurement system, applicable to all kind of exoskeletal devices.

This paper is organized as follows. In Section 2 we present the new distributed pressure sensor which is used as the base component in our sensory system. The working principle of the pressure sensor is presented, and a full characterization is given. Section 3 presents the new sensory apparatus to monitor the interaction on an exoskeleton connection cuff. The system is calibrated on four healthy subjects under static and dynamic condition. Section 4 presents an example of use of this new system to monitor the interaction pressures during a gait training task. Finally, Section 5 draws our conclusions.

## 2. The Soft Tactile Sensor

### 2.1. Design

Our focus being the application of distributed force sensing to the monitoring of human-robot interaction, we developed a new distributed soft force sensor, loosely based upon an artificial tactile technology developed in our laboratories, the Skilsens technology [22,23]. Our sensor (which we will refer to as “pressure sensor”, “Skilsens pad” or simply “pad”) is made of an array of sensitive elements based on a mechano-opto-electronic transduction principle. Each sensitive element is composed of a light emitter and of a light receiver, and the whole sensor is covered by a soft silicone shell. Besides covering the electronics and providing structural rigidity to the pad, the shell is directly involved in the transduction principle. A sketch of the single sensitive element is shown Figure 1(a).

A printed circuit board (PCB) houses a light emitter (an InGaN chip technology, high luminosity green LED, OSA Opto Light GmbH, Köpenicker Str. 325/Haus 201, 12555 Berlin, Germany) emitting light along the longitudinal direction, and a photodiode (an analog ambient light opto-electronic transducer with current output, Avago Technologies Ltd., 1 Yishun Avenue 7, Singapore), which gets the light from the side. When a load is applied on the sensor, it deforms its structure, which occludes the light path from the transmitter to the receiver, and reduces the light which reaches the photodiode, changing its current output. Each sensitive element has a dynamic, non-amplified range of about 0.2 Volts, with an output impedance of 22 kΩ. The signals are acquired using a 32-channels ADC board, with a sampling frequency of 2 kHz, and digitally filtered with a fourth-order Butterworth filter with a cutoff frequency of 40 Hz. The acquisition and filtering routines were implemented using NI Labview 2009 (National Instruments Corporation, Austin, TX, USA).

The size of the sensor was chosen based on the application (described later in Section 3). The length of the sensor was fixed to 60 mm, based on the height of the belt used to connect the user to the robot, and its width to 20 mm. With this size, as will be detailed later, the sensor does not interfere significantly with the flexibility of the belt onto which it will be attached. Since the sensor extends primarily along its length, the sensitive elements were positioned in a single row. A number of eight sensitive elements turned out to be the best compromise between increasing the spatial resolution (along the length of the sensor), and decreasing the optical interference between neighboring sensitive elements. Figure 1(c) shows the final appearance of the sensor. The mechanical stiffness, and therefore the maximum measurable force of the pad are mainly determined by the material and by the structural properties of a transversal section of the sensor.

**Figure 2.**
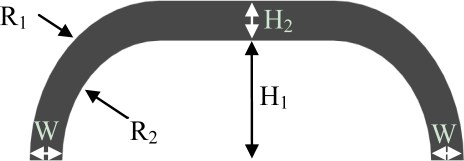
Cross section of the Skilsens pad. Highlighted are the internal (R_2_) and external (R_1_) radii, the upper thickness (H_2_) and lower thickness (W), and the inner height (H_1_).

The transversal section of the sensor is shown in Figure 2, and the main parameters of the section which determine the overall stiffness are highlighted. The section is defined by five geometrical parameters: the internal height (H_1_), the thickness of the silicone on the upper part (H_2_), the thickness of the silicone at the basis of the pad (W), and the internal and external radii (R_2_ and R_1_) which connect the basis of the pad with its upper part. The value of these parameters, along with the choice of the material, had to be chosen in order to fit the pad to the force range requirements of the task discussed in this work.

An interaction force range requirement of 60 N, corresponding to an average pressure on the pad of 50 kPa, was chosen based on a series of preliminary experiments [24]. The material we used was a shore A 40 platinum-catalyzed silicone (Sorta Clear 40, Smooth-On, Inc., Easton, PA, USA), colored with a black pigment. This material was modeled using a nine parameter Mooney-Rivlin solid model, and characterized by Axel Products Inc. (Ann Arbor, MI, USA). The four main characteristics which affect the structural behavior of hyperelastic elastomers were tested [25,26]: pure tension (using a long, thin specimen and a Video Extensometer), pure shear (using a very wide specimen and a Laser Extensometer), biaxial stress (through radial stretching of a circular disc, and a Laser Extensometer) and volumetric compression (with a cylindrical specimen). All the four tests were performed under slow cyclical loads, to avoid the Mullin effect (changing structural properties during the first time the material is loaded). The maximum engineering strain for which the material was tested was 0.5. Details on how this procedure is carried on are given in [25].

To obtain the desired force range, we worked on the geometrical parameters of Figure 2, by performing a finite element (FE) analysis using ANSYS 12 (Ansys Inc., Canonsburg, PA, USA). The experiment we simulated consisted in a rigid flat body interacting with the sensor parallel to the PCB, pushing the silicone structure. While the cross section of the silicone cover is constant along the length of the sensor, we could not perform a 2-dimensional FE analysis. The two extremities of the structure, which “close” the structure onto the PCB, contribute greatly to the overall structural behavior of the cover, and it was not possible to neglect their contribution in the structural analysis. Being that the cross section at the edges is not constant [see Figure 1(c)], a 3D FE analysis was required.

**Figure 3.**
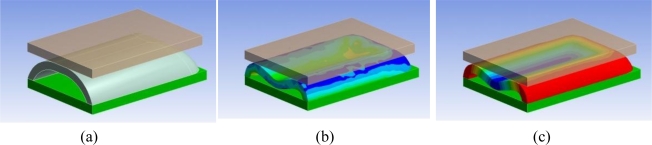
3D CAD representation of the simulated setup. In transparent brown, the rigid flat indenter, in grey, the silicone structure and in green, the PCB. **(a)** Undeformed structure. **(b)** Map of total stress. Blue corresponds to higher stress areas, green to lower stress areas. **(c)** Map of total deformation. In blue, the areas suffering a bigger deformation.

A representation of half of the simulated system is shown in Figure 3(a). The setup is composed of a rigid flat indenter, the silicone structure of the sensor, and the PCB. Exploiting two symmetries in the structure (along the longitudinal and transversal axes), we performed the FE calculations only on a quarter of the system (the simulation was run on half of the system shown in Figure 3). The contact between the flat indenter and the silicone was simulated as a rigid frictionless connection. This choice was made based on the difficulty of getting a reliable modeling of friction on hyperelastic materials [27]. The contact between the silicone structure and the PCB was modeled as a bonded connection. The nonlinearities in the simulation are related with the presence of a contact, and of the hyperelastic material model. We simulated the load by imposing a displacement of the indenter with respect to the PCB, and, for each deformation state, we evaluated the total stress state [an example is given in Figure 3(b)], the deformation state [example in Figure 3(c)], and the total force response of the structure. Our analyses on the silicone highlighted that the structure suffers a *sinking effect*, for which the pad gets more deformed in its central part then on its borders. This effect is shown in Figure 3(c) (with magnified deformation), and affects the transduction in two ways: on one side, it increases the deformation on the central part with respect to the borders, and thus increases its effect in terms of light occlusion; on the other side, it reduces the sensitive range of the sensor, because it decreases the force at which the silicone cover touches the PCB (and saturates the opto-electronic output).

**Figure 4.**
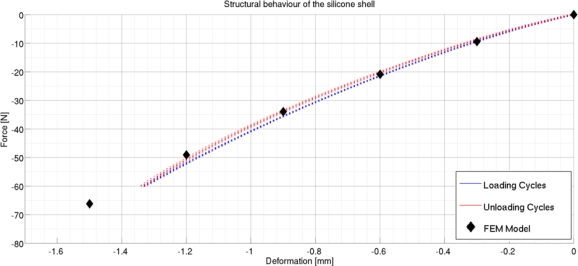
Force/Deformation characterization of the pad, after five loading-unloading cycles.

Taking these effects into account, we worked on the aforesaid five structural parameters to obtain the final design. Figure 4 reports the force/deformation behavior predicted by the FE analysis. It can be seen that the sensor was expected to reach the 60 N force range at a deformation of about 1.5 mm, which leads to saturation of the sensor’s output. The geometrical parameters of the final design are R_1_ = 6 mm, R_2_ = 6 mm, W = 3 mm, H_2_ = 3 mm and H_1_ = 4 mm. Starting from the final design parameters, the silicone shells are obtained by casting liquid silicone in a male/female acrylic mold. After polymerization, the silicone shell is glued on the PCB, completing the sensor production process.

### 2.2. Characterization

After production, each pad was characterized in its structural and electrical behavior. Both characterizations were obtained through a single procedure: a load was applied on the pressure sensor using a rigid flat body, to replicate the same setup of the FE simulation, and both the deformation and the voltage outputs were recorded. The characterization was performed using an INSTRON 4464 testing machine (INSTRON Inc, Norwood, MA, USA), equipped with a 1 kN load cell, and a rigid flat indenter. For each sensor, we performed five loading-unloading cycles, executed at a speed of 1 mm/min, to simulate quasi-static loads. Figure 4 shows the result of the structural characterization of the pad, where the total force on the sensor is compared with the total deformation. It can be seen that the behavior shown by the structure is close to the one predicted by FE simulations, and that, as desired, the maximum measurable load of 60 N leads to a deformation of about 1.5 mm, to give an average stiffness of 40 N/mm. The hysteresis of the structure is very small (about 3% of the full force range).

**Figure 5.**
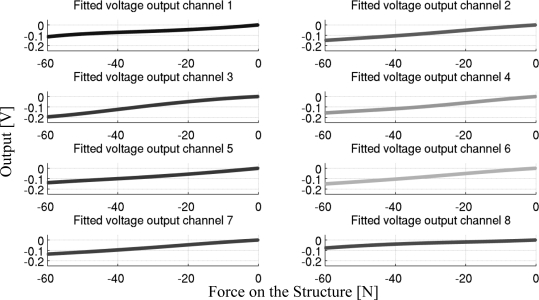
Voltage Output/Force behavior of the sensor. The fitted model is reported for each of the eight channels.

Moreover, we characterized the voltage output of the eight photodiodes as a function of the applied loading force. This characterization is necessary to make a one-to-one correspondence between the output voltages of the sensitive elements, and the force acting on the structure. This is required to estimate the pressure distribution on the sensor and the total force, as described in Section 2.3. Figure 5 shows an example output for the eight channels as a function of the applied loading force. It can be seen that the input/output relation for all the channels is smooth, with no critical nonlinearities, and a non-amplified gain of about 3.3 mV/N. While the output of most of the channel is fairly linear, we decided, for better accuracy, to fit the data with a 5-node, third order spline interpolator (the fitting was performed using Mathworks™ MATLAB®, and the Shape Language Modeling toolkit, Copyright (c) 2009, John D’Errico). The electrical characterization and data fitting constitutes the model of the sensor, and needs to be performed on each different pad, due to the variability of the light/voltage characteristic among different photodiodes, and also due to differences in the material properties itself. Therefore, we characterized each of the three sensors (see Section 3) used in this work separately. The goodness of fit is evaluated in Figure 5 and Table 1, which report the normalized RMSE and maximum percentage error of the signals collected on the loading-unloading cycles, compared to the fitted model. These results show that, for a given load, the voltage output of the sensitive elements is very repeatable, and that the effect of the structural hysteresis on the output accuracy is low.

### 2.3. Force and Pressure Distribution Estimation

To estimate the force and pressure distribution acting on the sensor from the eight voltage outputs, we implemented a simple estimation algorithm, which uses the model of sensor derived with the characterization described in the previous section. This algorithm is based on the assumption that the force and voltage of each of the eight sensitive elements is not correlated with that of the other neighboring elements. This is a simplification: the deformation of one element depends on the deformation of the neighboring elements because the silicone cover is a single structure. With this method, it is necessary to characterize the sensor only once, under uniform loading condition. The force estimation algorithm, however, does not make the hypothesis that the load is uniformly distributed, rather, it can be used (with variable performances) under all loading conditions. The algorithm works as follows:

1. the signals are filtered and de-offset;
2. the eight voltages are used as input for the force/voltage models (as per Section 2.2), to extract eight force values;
3. the eight resulting forces are averaged to determine the estimated force on the sensor;
4. (parallel to 3) the eight resulting forces are transformed in eight pressure distribution values (dividing by the surface of the pad).

**Figure 6.**
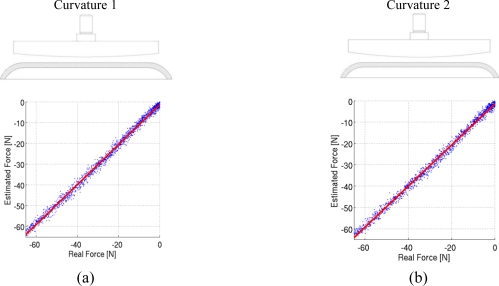
Force estimation results under non-uniform loading conditions **(a)** with a curvature of 3 m^−1^, **(b)** with a curvature of 5 m^−1^.

Due to the assumption made by this algorithm, the accuracy and measurement noise of the sensor depend on the loading condition, and need to be evaluated for each expected loading pattern. To give an example of the performances of the sensor, we tested it under two different nonuniform loading conditions. We developed two rigid indenters with a curve indentation face (two different curvatures, 3 m^−1^ and 5 m^−1^ were tested). We analyzed the performance of the sensors by applying loads in the range of 0 to 60 N, and by comparing the output of the force estimation algorithm with the recording of a load cell. The experimental setup was the same used in the calibration phase, as 5 loading-unloading cycles where performed at a constant speed of 1 mm/min. Figure 6 reports the estimation results for the two conditions, with a sketch of the indenter used for the purpose. The blue dots represent the estimation of the algorithm, and the red line a linear fitting of the estimates. It can be seen that non-uniform loads introduce two sources of error in the estimate. Table 2 reports the normalized and absolute RMSE of the measurements to evaluate measurement noise, as well as the systematic error of the sensor. The normalized RMSE was calculated comparing the estimated force with the load-cell force, and normalizing with the full scale force of 60 N. The systematic error was evaluated by linearly fitting the estimates of the pressure sensor, and by comparing the slope of the fitted curve with the ideal steepness of 1 (which corresponds to a measure with no systematic error). The maximum error introduced by this systematic effect on the measure is of about 2 N, which is well below the measurement noise as evaluated by the RMSE (which can reach 5 N).

The conditions we tested certainly do not represent the load distribution that will act on the sensors during normal operation. They prove however that the performances of the sensor are dependent on the load distribution, and that a calibration is necessary with the loading conditions expected when using the sensor. For this reason, in the following Section we will perform a calibration of the sensor applied to the exoskeleton, with four different subjects, to evaluate the measurement noise and accuracy in the real-world application.

## 3. Sensing Human-Robot Interaction in Lower Limb Exoskeletons

This Section deals with the application of the distributed pressure sensor we developed to the actual interaction-measurement sensory apparatus. As stated before, the performance of the sensor depends on the loading condition. Therefore, the only way to test the sensors in a reliable way is to apply the apparatus to the exoskeleton, and calibrate the outputs of the sensors with a reliable interaction measure, such as that of a six-axes load cell. For this reason, we applied our system to the connection cuff of a lower-limb exoskeleton (Section 3.1), and tested it with static loads (Section 3.2), dynamic loads (Section 3.3), and during a prototypical rehabilitation task (Section 4).

All the experiments were performed on four male healthy subjects (age 28 ± 3 years, weight 74 ± 3 kg, height 174 ± 1 cm). The subjects we choose are clearly not representative of the population meant for the rehabilitation protocols for which this exoskeleton is used. However, our objective was not to replicate or represent a typical rehabilitation protocol and its population, but rather to test a measurement system on a small pool of subjects. We selected subjects of similar build and size to reduce variations in the attachment points positions, and cuff size, and therefore to give comparable calibration results (in terms of preloading and force fraction unloaded on the pads, see Section 3.2).

### 3.1. Materials and Methods

**Figure 7.**
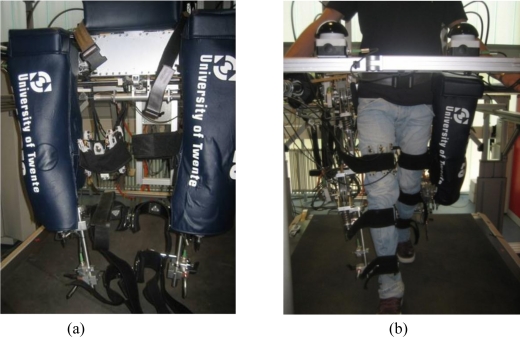
**(a)** The LOPES gait rehabilitation exoskeleton. The right leg upper cuff is equipped with the sensory system. **(b)** The LOPES exoskeleton during operation.

**Figure 8.**
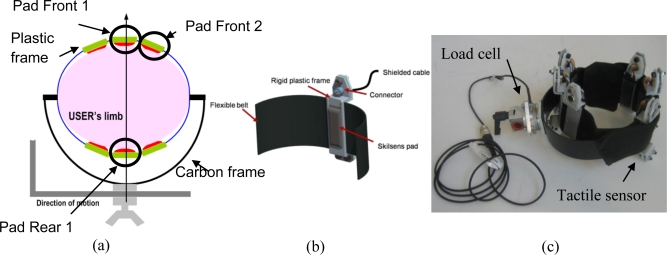
**(a)** Transversal section of the sensorized fastening belt. **(b)** 3D sketch of the sensor housing. **(c)** Experimental setup for the cuff used in the experiments.

Our case study is the LOPES gait rehabilitation and assessment robot [9], shown in Figure 7(a). LOPES is an 8-degrees-of-freedom powered exoskeleton, which can assist the gait of the user with three actuated degrees of freedom for each leg, two at the hip, and one at the knee, and with additional translational degrees of freedom to move (or fixate) the pelvis in the coronal and transverse planes. The LOPES joints are powered by series elastic actuators [28] that can be controlled either in torque mode, or using a virtual-mode impedance control [9] which allows the definition of an attraction trajectory, and a virtual spring constant. The user is strapped and linked to the exoskeleton through three attachment points for each leg: one on the upper leg, and two in the lower leg. As Figure 7(a) and Figure 7(b) show, our sensory apparatus was applied to the right upper-limb cuff. The cuffs used in this robot are manufactured by Hocoma (Hocoma AG, Industriestrasse 4, CH-8604 Volketswil, Switzerland), and are made of a rigid carbon fiber frame directly connected to the robot link through a steel bar, and of a flexible belt which can be fastened to the leg. Figure 8(a) shows a sketch of a transversal section of the cuff. The forces are transmitted by the robot to the connection cuff through the steel bar, and then to the belt strapped around the user leg, which is supported by the carbon fiber frame. This cuff is also used in other exoskeletal robots, most notably the Lokomat [5] for which the cuff has been originally designed. Similar solutions, consisting of a rigid frame and of a flexible belt, are used in other lower-limb [4,13] and upper limb robots [10,12].

The solution proposed in this work, while being specific for this cuff, can be easily extended to similar attachment systems. This configuration, sketched in Figure 8(a), consists in putting a number of sensitive pads in between the flexible belt and the user’s limb. In order to house the Skilsens pads, and keep them fixed on the belt, we designed a rigid plastic frame, shown in Figure 8(b), whose bulk is entirely on the outside part of the cuff. This way, only the silicone structure of the sensor is in contact with the limb, to preserve the interaction comfort. This frame, which also houses the connector for the compound signal/power cable, allows to easily increase or decrease the number of sensors distributed over the belt, as well as to quickly change their position. As stated in Section 2, the width of the sensors (and, consequently, the maximum number of pads which can fit in a single belt) was chosen to be 20 mm (with an additional encumbrance due to the frame of 6 mm). This allows a good force measurement resolution along the belt (depending on the circumference of the leg, up to 10 to 12 sensors can fit on the cuff), and does not interfere significantly with the flexibility of the belt.

In all the experiments performed in this work, only the thigh connection cuff was sensorized, with six sensitive elements, three in the front, and three in the back, as shown in Figure 8(c). In addition to that, the cuff attachment point was sensorized using a 6-axes load cell (ATI Mini45, ATI Industrial Automation, 1031 Goodworth Dr., Apex, NC 27539 USA) to provide a reliable measurement reference to be used for calibration and validation of the system. In this work, we acquired only the signals relative to three pads, two in the front, and one in the rear of the cuff. These sensors are highlighted in Figure 8(a).

### 3.2. Calibration—Static Loading

A first calibration was performed to evaluate the precision and accuracy of the sensor with static loads, with the sensor in contact with the user. This characterization was performed to verify the effectiveness of the sensor during normal working conditions, which differ from the tests performed in Section 2.2 because:

- the sensor is loaded with a pressure distribution and deformation profile different from the controlled loading conditions used in the sensor characterization;
- the sensor is in direct contact with the user’s thigh, which has an irregular shape and differs from one subject to another;
- the position of the sensors determine how the interaction force distributes along the belt, and, therefore, the fraction of interactive force which is unloaded on the pad. This changes among different subjects and sensors;
- the sensor may move slightly during normal operation, and it is not known how much this will affect the measurement.

**Figure 9.**
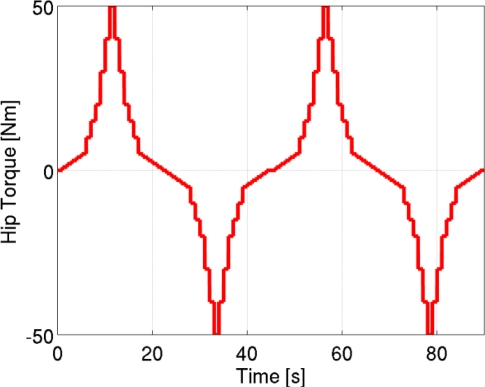
Hip torque steps performed during the characterization.

The calibration was performed on each subject, with the right leg in the vertical resting position, with the foot fixed to the ground. The subjects were asked not to move, and incremental torque steps ranging from −50 Nm to +50 Nm were applied to the hip joint, as shown in Figure 9. The resulting interactive force transmitted to the thigh was measured using the load cell. At the same time, the pressure distribution and total force acting on each pad was estimated using the algorithm described in Section 2.3. All the data were acquired in static conditions, neglecting all transient effects.

By comparing the output of the load cell and the force acting on the sensor under static conditions, we can evaluate for each sensor, and each user:

- the *fraction* of interactive force unloaded on each pad. This factor is fixed, and does not change as long as the pad does not move on the belt: therefore, we expect a linear relation between the total interactive force, and the measured force;
- the *preloading* force, due to the fastening of the belt, that acts on the pads. This value corresponds to the measured force when no interactive force is present.

**Figure 10.**
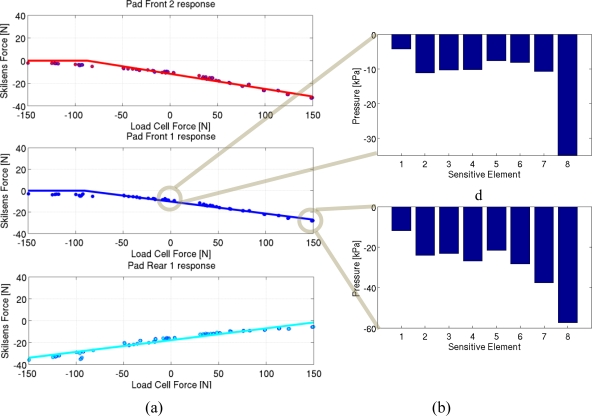
Static characterization results: **(a)** Force on the three pads, compared with the force recorded by the load cell. **(b)** Pressure distribution on the sensor at zero and peak interaction. Channels are ordered from top (1) to bottom (8).

The result of the calibration, which is shown in Figure 10(a), as an example, for Subject 1, is the comparison of the total interactive force (measured by the load cell, on the x-axis) with the force acting on each pressure sensor (Skilsens force, y-axis). Only compression forces (negative in our sign convention) can be measured by our pressure sensor. The linear fitting is therefore split into two line segments: one for negative values of the pressure sensor (*i.e.*, in the working range of the sensor), and one which represents the range in which the pad is unloaded (and its measured force is 0).

Three factors are highlighted by the static calibration. The *preloading* acting on each pad corresponds to the y-intercept of the fitted curve, which is the force on the pad when no interactive force is applied. This value (and the corresponding pressure distribution) is equivalent to the baseline force caused by the fastening of the belt on the thigh. The slope of the curve represents the *fraction of interactive force* unloaded on each pressure sensor. This percentage value determines how the total interactive force at the upper-limb is distributed among different areas of the cuff. Therefore, it determines the pressure distribution along the length of the belt. Finally, the x-axis intercept of the linear curve delimits the *range of forces measured by the sensor*. For each subject the results of the static calibration (along with error data) are shown in detail in Table 3.

In addition, static calibration can also give an idea on how pressure is distributed along the width of the sensors during static loads. This is useful, for example, to evaluate the baseline pressure acting on the thigh when no loads are applied by the exoskeleton, or to evaluate pressure distribution when peak loads are transmitted. Figure 10(b) shows, as an example, the pressure distribution on the eight elements of one of the sensors at zero interaction (upper plot) and peak interaction (lower plot).

### 3.3. Dynamic Loading

The same experimental setup of the static calibration was replicated on the four subjects to verify the behavior of the sensors under dynamically-varying loading conditions. To apply a dynamic load on the user, a torque chirp (frequency range: 0–3 Hz, total time: 100 s, amplitude: 30 Nm, offset: −25 Nm) was commanded to the LOPES hip joint, while the subject stands with the foot fixed on the ground and is asked not to move. The frequency range was limited to 3 Hz due to torque control bandwidth limits of the exoskeleton (which uses a series elastic actuation), and the offset was set to keep the interaction force along the same direction.

The frequency range was chosen to apply strongly time-varying interaction forces at the limb, while not being too demanding both for the user and the robot. Higher frequency loads would have been strongly uncomfortable for the subjects, and also demanding for the structure and frame of the robot. Compared to the static characterization, the main difference that we are investigating is the presence and quantification of dynamic attenuation effects on the force measurement of the sensors.

**Figure 11.**
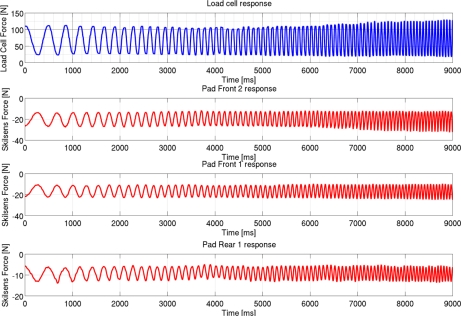
Dynamic characterization results: Force acting on the three pads, compared with the force recorded by the load cell.

Figure 11 shows the interaction force recorded by the load cell, and by each of the three pads, during the dynamic characterization. The measurements are shown, as an example, only for one subject (Subject 1), but results were equivalent on the other tests. It can be noted that, differently from the hip torque, the hip interaction force measured by the load cell is not a perfect chirp. This is due to dynamic effects induced by vibrations and small movements of the robot and of the subject during the task.

### 3.4. Discussion on Static and Dynamic Characterization

The result of the calibration under static condition proves that the sensor can effectively be used to monitor the user-exoskeleton interactive force during normal operation. According to data reported in Table 3 for all subjects, and all sensors, a constant fraction of interactive force is unloaded on each pad, ranging from 17–21% for the most loaded pads, to 7–8% for the less loaded pads. This fraction depends on the position of the sensor, as well as on the size of the thigh, so it changes across different sensors, and subjects. This linear relation allows estimating the total interactive force on the attachment points, in the working range of the tactile sensor. Multiple pads estimates can thus give redundancy, and therefore better accuracy, to the measurement of the resulting interaction, making it a viable substitute for a load cell. Moreover, this calibration allows to evaluate how the interaction is distributed along the belt.

Furthermore, the calibration reveals the preloading force acting on each sensor. This value was also highly variable across subjects, since the preloading force depends on where and how tightly the cuffs are fastened to the user’s thigh. Accordingly, it can be seen that the preloading forces are different among pads attached to the same cuff. The preloading values range from 17–18 N for the most tightly fastened cuffs, to 4–5 N for the most loosely fastened. Giving a measurement of the preloading force, our sensor allows the therapist to fasten the cuffs in a repetitive and reliable way.

Our pressure sensor is sensitive only to compression loads. The preloading force induced by the fastening causes a pre-compression of the pad. Therefore, depending on the direction of the interaction applied by the exoskeleton, the pad will either be compressed or uncompressed. For this reason, each pad is sensitive to interaction forces in both directions.

With the preloading forces and interactive torques applied in our experiments (comparable to those applied during gait rehabilitation tasks [9]), it can be seen that full range saturation is never reached, and that, depending on the subject, the pads can have a good bi-directional interactive force range.

The dispersion of the static characterization data for each pad compared to the linear fitting can be imputed to different reasons: the sensor noise; the errors due to the force estimation algorithm; and small movements of the pad with respect to the thigh during the execution of the task. Indeed this last effect can also slightly change, locally, the preloading and the slope of the curve.

The sensor also allows one to determine how pressure is distributed along the width of the belt (corresponding to the length of the sensor). Figure 10(b) is an example of how pressure can be distributed unevenly not only along the length of the cuff, but also along its width. The sensor allows to extract eight pressure distribution values. This eight measures can be used either directly to detect the load distribution, or to extract a single value of interest, like the total loading force [as in Figure 10(a)], the average pressure, or the peak pressure (which is of particular interest when evaluating comfort).

The dynamic characterization proves that the sensors do not suffer any significant effect during dynamic loading conditions, at least in the range of loads which could be provided by the LOPES exoskeleton. The results showed in Figure 11 and Table 4 show that, as for the static condition, a constant fraction of the interactive force is unloaded on each sensor. This fraction does not depend on the frequency components of the loading pressure, showing that no significant effect is introduced by the dynamic loading condition.

These results show that our sensory apparatus can be effectively used to monitor human robot interaction in a lower limb exoskeleton. In controlled conditions, it has been shown that a constant fraction of interactive force unloads on each sensitive element, both in static and dynamic loading conditions. For this reason our sensory system can be used to evaluate the resulting human-robot interaction force, providing a redundant and therefore highly reliable measurement. More than that, our sensory system allows evaluating how the interactive forces are distributed over the contact area on the user’s limb. Compared to single point measures, therefore, our system provides an objective mean to evaluate the interaction comfort (in terms of local pressure on the limb), and allows to quantify the fastening force (by monitoring the preloading of each pad) on the belt. In this Section, we performed a characterization of the sensory system by comparing the output of each tactile sensor with that of a load cell. Similar results could have been obtained by comparing with a different interaction force estimate, obtained through measurements of the interaction torque. For example, model-based interaction torque estimates, or direct measurements from a reliable torque source, could have been used.

In the prototype presented in this work, only a fraction of the contact area was covered with sensors. This means that, while the sensors do not move along the belt [they are fixated as shown in Figure 8(c)], it is certainly possible that the relative position of the thigh and the sensors change due to small slippages. This may happen if the belt is not correctly fastened on the thigh, or if the belt size is too big compared with the circumference of the thigh. During our experiments, the fastening force was sufficient to fixate the relative position of the belt and the thigh. This is proved by the fact that a constant fraction of interactive force is unloaded on each sensor (Figure 10). If a sensor moves to a different part of the user’s thigh, a different fraction of force unloads on its surface. Therefore, the calibration is invalidated, and an error is made when using the measure of the sensor to estimate the total interaction force. In a final prototype of this sensory system, the full interaction area will be covered, and all the interaction will be unloaded on the sensors, thus eliminating this problem at its root.

## 4. A Case Study: Walking in a Simulated Viscous Field

As a final assessment of our sensory apparatus, we present an analysis of the interaction pressure distribution during a gait training task. In this experiment, a subject wearing the exoskeleton, with the same sensorization described in the previous Sections, was asked to walk on a treadmill at a constant speed of 4 km/h. Two different conditions were analyzed. In the first one, the exoskeleton was controlled in zero-torque mode [29], where it operated as transparent as possible. In the second condition a viscous field of 10 Nm/rad·s^−1^ was applied at the hip joint, to simulate a gait training task. Each condition was kept for about 250 gait cycles (about 2.5 min).

Alongside the kinematic data, as before, we collected the pressure data of each sensor, as well as the total interaction force measured by the load cell. All the recorded data was averaged over the gait cycles, to give a clear picture of the general tendency of the interaction forces.

Figure 12 reports the results of the acquisition, the common x-axis representing the percentage of the gait cycle. Figure 12(a) reports the average pressure and total force unloaded on each pad. Figure 12(b) shows an example of how pressure distribution varies during the gait cycle (frontal pad 2 is shown). The beginning of the cycle (0–100%) corresponds to the foot impact on the ground. The stance phase ranges from 0 to about 50–60% of the cycle, where the toe-off takes place. The remaining part of the cycle (60–100%) corresponds to the leg swing phase.

Another interesting behavior to be noted relates to pressure over the rear pad during the gait. Comparing the output of the load cell, which represents the overall interaction force, with that of the pads, it can be seen that, while the two frontal pads have the same tendency of the overall interaction force (negative, in the 0–30% range, with a surge at the beginning of the swing), the rear pad shows a completely different behavior. In the central part of the stance phase (10–50% of the gait cycle), a peak in the local pressure on the rear part of the thigh is detected by the tactile sensor. A similar, smaller surge can be seen even on the second frontal pad, in the opposite direction. These peaks are probably due to the co-contraction of the leg muscles during the stance phase, and to the consequent change in the shape and size of the thigh. These peaks of local pressure do not correspond to a decrease in the overall interaction force, and *would not have been detected by using the load cell alone.* These evaluations are just an example of how it is possible to quantify the local interaction pressure distribution during a gait training task using a rehabilitation robot, and how the information conveyed by this distributed sensory apparatus is richer than that of a single-point measurement.

## 5. Conclusions

In this work we propose a new method to provide a distributed measure of the interaction pressure on the human-robot interface in exoskeletons. The proposed system is based on a distributed measure of the pressure over the user-robot contact area, obtained by applying a distributed pressure sensor between the user and the exoskeleton. A prototype of the sensory system was developed, and tested on a gait rehabilitation robot, the LOPES lower-limb exoskeleton, on four healthy subjects. The sensory apparatus proved to give accurate, redundant and reliable measurements of the interaction force. On top of that, it allows monitoring the local pressure distribution on the user’s limb, providing an objective mean to evaluate the local stress on the user, and therefore the comfort of interaction

This sensory system represents a first step towards the development of a general-purpose, flexible and adaptable distributed interaction measurement system, applicable to all kind of exoskeletal devices. Such a system could represent a valuable tool for monitor the local stress on the users’ skin, allowing to change and tune the control of the robot to avoid excessive localized pressure, and also to monitor how interaction is distributed during interaction. This could be of particular interest in fields where ergonomy of the interaction is critical, like rehabilitation robotics.

While this work focused on the analysis of pressure distribution, this sensory system could also be used to detect deformations of the user tissues, especially in robots which use undeformable orthotic physical interfaces (flexible belts accommodate the deformation of the muscle by deforming themselves). It could be interesting to verify whether this could constitute a viable, low-cost alternative to more direct and invasive measurements of muscular activity, such as surface EMG.

## Figures and Tables

**Figure 1.**
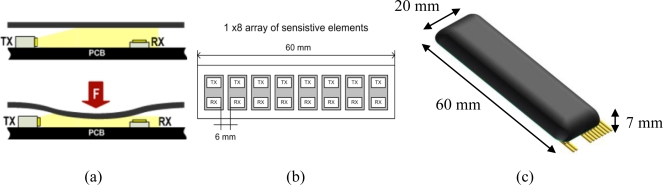
**(a)** A cross section of the sensor, showing the light transmitter (TX) and receiver (RX) **(b)** Position of the eight sensitive elements. **(c)** Overall view of an 8-channel Skilsens Pad.

**Figure 1.**
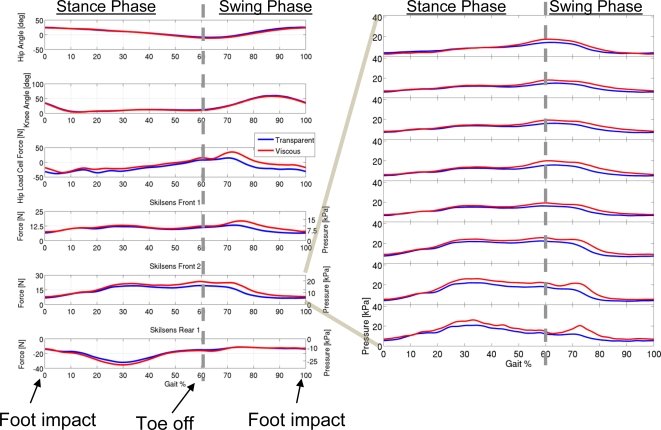
**(a)** Force on the three pads compared with the measurement of the load cell, during a walking task. **(b)** Pressure distribution on one of the frontal pads.

**Table 1.** Normalized root mean square error of the signal, compared with the fitting, and maximum percentage error (relative to the full scale range), of the eight channels for each of the three pads used in this study.

	
	**Normalized RMSE [V] | Maximum % Error [V]**															

	**Channel 1**		**Channel 2**		**Channel 3**		**Channel 4**		**Channel 5**		**Channel 6**		**Channel 7**		**Channel 8**	
**Pad 1**	1.1%	3.0%	1.1%	4.0%	0.9%	2.5%	0.9%	4.0%	0.8%	3.5%	0.9%	4.0%	1.0%	3.5%	1.0%	3.5%
**Pad 2**	1.0%	4.0%	1.0%	3.0%	1.0%	4.0%	0.8%	3.0%	0.7%	2.5%	1.2%	4.1%	0.7%	2.6%	0.8%	3.0%
**Pad 3**	2.1%	5.0%	2.1%	4.5%	1.5%	6.5%	1.1%	4.5%	1.3%	4.5%	1.2%	3.0%	1.0%	3.0%	0.8%	3.5%

**Table 2.** Normalized and absolute RMSE of the estimated force, and systematic measurement error compared with the load-cell force, for the three pads under the three loading conditions.

	
	**Curvature 1**		**Curvature 2**	
	**Systematic % Error**	**RMSE [N% (N)]**	**Systematic % Error**	**RMSE [N% (N)]**
**Pad 1**	3.8%	7.2% (4.3)	3.4%	7.5% (4.5)
**Pad 2**	3.7%	2.7% (1.6)	3.3%	2.7% (1.6)
**Pad 3**	3.8%	7.8% (4.7)	3.4%	8.5% (5.1)

**Table 3.** Fraction of the interaction force unloaded on each pad, and preloading force acting on each sensor, for the four subjects.

	
	**Linear Fitting**					
	
	**Subject 1**			**Subject 2**		
	**Force %**	**Preloading [N]**	**RMSE [N]**	**Force %**	**Preloading [N]**	**RMSE [N]**

**Front pad 1**	13.3	11.6	1.31	17.8	4.5	1.06
**Front pad 2**	11.2	10.1	0.89	10.4	16.6	2.47
**Rear pad 1**	10.74	17.7	2.05	7.5	7.8	1.68

	**Subject 3**			**Subject 4**		
	**Force %**	**Preloading [N]**	**RMSE [N]**	**Force %**	**Preloading [N]**	**RMSE [N]**

**Front pad 1**	11.6	9.38	1.87	21.5	8.0	2.15
**Front pad 2**	7.9	18.3	2.83	12.7	7.5	1.78
**Rear pad 1**	17.3	13.0	0.84	14.8	5.0	1.05

**Table 4.** Fraction of the interaction force unloaded on each pad, and preloading force acting on each sensor, for the four subjects during the dynamical tests.

	
	**Subject 1**		**Subject 2**		**Subject 3**		**Subject 4**	

	**Static Gain (Force %)**	**Attenuation at 3 Hz [dB]**	**Static Gain (Force %)**	**Attenuation at 3 Hz [dB]**	**Static Gain (Force %)**	**Attenuation at 3 Hz [dB]**	**Static Gain (Force %)**	**Attenuation at 3 Hz [dB]**
**Front pad 1**	15.2	−0.1	20.4	−0.1	17.8	−0.1	17.9	−0.1
**Front pad 2**	12.3	−0.1	19.8	−0.1	16.2	−0.1	12.4	−0.1
**Rear pad 1**	13.9	−0.3	6.6	−0.4	8.3	−0.3	3.4	−0.4

**Table 5.** Peak pressure and force on the pads, in the two conditions shown in Figure 12.

	
	**Preloading**		**Peak Pressure [kPa]**		**Peak Force [N]**	
	**Pressure [kPa]**	**Force [N]**	**Transparent**	**Viscous**	**Transparent**	**Viscous**
**Front pad 1**	10.4	12.5	11.0	14.0	13.2	16.8
**Front pad 2**	13.4	16.1	16.1	23.6	19.3	23.5
**Rear pad 1**	15.2	18.2	26.6	29.5	32.0	35.4
